# Simulating the Feasibility of Using Liquid Micro-Jets for Determining Electron–Liquid Scattering Cross-Sections

**DOI:** 10.3390/ijms23063354

**Published:** 2022-03-20

**Authors:** Dale L. Muccignat, Peter W. Stokes, Daniel G. Cocks, Jason R. Gascooke, Darryl B. Jones, Michael J. Brunger, Ronald D. White

**Affiliations:** 1 College of Science & Engineering, James Cook University, Townsville, QLD 4811, Australia; peter.stokes@my.jcu.edu.au (P.W.S.); ronald.white@jcu.edu.au (R.D.W.); 2 Department of Medical Physics, Townsville University Hospital, Townsville, QLD 4814, Australia; 3 Research School of Physics and Engineering, Australian National University, Canberra, ACT 0200, Australia; daniel.cocks@gmail.com; 4 Synchronous Technologies PTE LTD, 6 Raffles Quay, #11-07, Singapore 048580, Singapore; 5 College of Science & Engineering, Flinders University, Bedford Park, SA 5042, Australia; jason.gascooke@flinders.edu.au (J.R.G.); darryl.jones@flinders.edu.au (D.B.J.); michael.brunger@flinders.edu.au (M.J.B.); 6 Institute of Actuarial Science and Data Analytics, Faculty of Business and Management, UCSI University, Kuala Lumpur 56000, Malaysia

**Keywords:** cross-section, electron, liquid microjet, machine learning, Monte Carlo

## Abstract

The extraction of electron–liquid phase cross-sections (surface and bulk) is proposed through the measurement of (differential) energy loss spectra for electrons scattered from a liquid micro-jet. The signature physical elements of the scattering processes on the energy loss spectra are highlighted using a Monte Carlo simulation technique, originally developed for simulating electron transport in liquids. Machine learning techniques are applied to the simulated electron energy loss spectra, to invert the data and extract the cross-sections. The extraction of the elastic cross-section for neon was determined within 9% accuracy over the energy range 1–100 eV. The extension toward the simultaneous determination of elastic and ionisation cross-sections resulted in a decrease in accuracy, now to within 18% accuracy for elastic scattering and 1% for ionisation. Additional methods are explored to enhance the accuracy of the simultaneous extraction of liquid phase cross-sections.

## 1. Introduction

The interaction of a low-temperature plasma with liquids is fundamental for numerous new and emerging technologies, finding applications in important domains, including environmental remediation, and the synthesis of nanomaterials and medicine [1,2,3,4,5,6,7,8,9]. The goal of high-level optimisation, of the efficacy and selectivity, of these and future generation plasma–liquid applications depends on, among other things, a detailed understanding of the underlying fundamental nanoscale physics and associated predictive modelling, underpinned by accurate and complete transport theory and cross-sections. The key driver to these applications is the role of electrons (and other radical species) at the plasma–liquid interface, but despite their central role, electron-induced transport and processes at the interface are not well understood [10].

Developing our understanding of electron transport into and within liquids is critical for enhancing the predictive power of plasma–liquid models. Fundamental to transport theory, which governs the motion of electrons into and within such environments, are complete and accurate sets of electron impact cross-sections. While there is a wealth of knowledge of electron impact cross-sections in the gas phase (e.g., databases such as LXCat [11,12]), the same is not true for the liquid environments. The scattering and transport theory of pre-solvated electrons in non-polar liquids is reaching some level of maturity (see the ab initio treatment by the authors [13,14]), however this is not the case for polar liquids. An existing Monte Carlo simulation of low-energy electrons in liquid water [15] ignored many physical processes, that are relevant to developing appropriate scattering theory. Furthermore, current models use cross-sections calculated in the gas phase, or through electron reflection measurements from amorphous ice [16]. The absence of liquid phase data represents a large knowledge gap in the literature, and experiments proposed in this manuscript aim to address, at least in part, this knowledge gap.

Here, we propose a new experimental technique that extends gas phase beam experiment methodologies [17] to include electron scattering from liquid surfaces, through the use of liquid micro-jets (LµJs). A constant replenishment of liquid, a small surface area, and a laminar flow enable the scattering of particles from a smooth and stable liquid surface, whilst operating in a vacuum environment. Until now, LµJ investigations have focused primarily on photon and photoelectron scattering [18,19,20,21,22,23,24,25,26,27,28,29,30]. In this work, we introduce electron scattering from LµJs and investigate the feasibility of developing effective electron–liquid cross-sections from the measured electron energy loss spectra (EELS). Unlike gas phase experiments, which are designed to ensure single scattering processes only, the proposed experiment is necessarily multi-scattering, with electrons being scattered from the surface and bulk atoms/molecules. The connection of this with traditional (multiple collision) swarm experiments [31,32,33,34] is thus clear.

Swarm experiments have proven to be critical in the development of accurate cross-section sets [17]. In electron swarm experiments, electrons are driven through a gaseous (or liquid) medium by an applied electric field, and macroscopic descriptors such as current, drift velocity, and diffusion coefficients are determined. Simulation techniques then evaluate the accuracy and self-consistency of the cross-section sets through comparisons of the transport coefficients. The same techniques can be used, in principle, to iteratively improve or develop cross-section sets [35,36,37,38,39,40], although the question of degeneracy in the cross-section set remains open, and other means are usually required to minimise this. Recent studies have shown promising applications of neural networks (NN), toward improving cross-section sets through swarm transport data [41,42,43,44,45,46]. Utilising a similar NN methodology, along with energy loss spectra from a LµJ experiment, we propose the generation of cross-section sets for electrons within a liquid environment. To facilitate this, a benchmarked Monte Carlo simulation technique, for simulating electron transport in liquid environments [47], has been developed and implemented for this study, with extensions to calculate EELS arising from the scattering of electrons from an LµJ.

The paper is organised as follows. In Section 2, we provide a summary of LµJs and their implementation in the proposed experiment. In Section 3, we outline the Monte Carlo (MC) simulation method utilised here and discuss the characterisation of bulk liquid and interfacial effects. The characteristic signatures of the various multiple scattering elements on the EELS are also discussed. In Section 4, we use the developed Monte Carlo software to train a neural network to predict neon’s elastic and ionisation cross-sections as a proof-of-concept. Finally, in Section 5, we provide some concluding premarks.

## 2. The Proposed Electron–Liquid Micro-Jet Scattering Experiment

### 2.1. Liquid Micro-Jets

Liquid micro-jets were first developed to overcome the limitations of probing liquids under vacuum conditions, which are often required by scattering experiments [48]. Due to the high vapour pressures found in liquids, additional treatment is required to ensure a stable surface within vacuum conditions, and hence the developments of the liquid jets. In our proposed experiment, a replenishing and sufficiently thin liquid source, exhibiting laminar flow, facilitates a smooth and stable scattering surface within a vacuum environment [49].

Siegbahn and Siegbahn [48] first utilised a replenishing 0.2mm diameter liquid jet, and scattered electrons from that source to measure binding energies in formamide ($\mathrm{HOCN}H_{2}$). In vacuo experiments were initially limited to low vapour pressure liquids, to avoid immediate freezing and/or evaporation. For high vapour pressure liquids such as water, a further reduction of exposed surface area is required to maintain a free vacuum surface [20]. To this end, significantly smaller (∼10µm) liquid micro-jets were first developed to measure the velocity distributions of vapour molecules, while ensuring inter-molecular collisions were minimised [49]. Extending this, a focusing gas surrounding the nozzle was implemented to increase the length and decrease the diameter of the jet substantially [50,51,52]. Flat LµJs were also developed from the collision of two micro-jets, which results in a sheet-like scattering surface [53]. Additionally, cryogenic jet systems enable the use of super-cooled liquids with jet diameters reaching 1µm [54,55]. It is clear that a wide array of configurations are achievable, and can be tailored towards the desired experiment.

Until now, applications of the aforementioned LµJ designs include an array of photoelectron spectroscopy experiments, to measure properties such as binding energies and hydrogen bonding [18,19,20,21,22,23,24,25,26,27,28,29,30]. Other LµJ experiments include mass spectrometry [56], enhanced X-ray production [57,58,59,60], X-ray emission/absorption, to probe electronic structure and molecule orientation [61,62,63], to study the molecular dynamics of evaporation [64], electrokinetic power generation [65,66], and drug delivery alternatives [67]. For further general reading, one is directed to the summary papers cited here [23,68,69].

Scattering simulations, such as the MC code developed at James Cook University, rely on the characterisation of the LµJ features. Liquid dynamics studies of LµJs found jet length stability on the order of millimetres. In one study [70], source widths of 10, 20 and 50µm, along with velocities ranging between 25 and $150\;{\mathrm{ms}}^{-1}$, were trialled for each diameter. A limited laminar flow was achieved with jet lengths between 1 and 15mm. After emergence from a circular nozzle, the jet undergoes contraction to a final cross-sectional size due to surface tension forces [71]. Typical contraction values were found to range between 90% and 60% [70] of the original source width, after which capillary forces break the jet into droplets through a tendency to try and reduce surface energy [71].

Within the liquid, charge build-up, and the existence of a streaming potential, due to electrokinetic charging, can hinder the accurate simulation of charged particle transport. A streaming potential is produced when a pressurised liquid is forced through the nozzle, which disrupts the electric double layer created between the liquid and the inner wall of the nozzle [72,73]. For liquid water, the magnitude of this streaming potential exceeds 60V depending on the jet velocity and diameter [19,73]. The addition of an electrolyte has since been shown to minimise this effect [73]. Additionally, the production of electrons within an insulating medium such as water will produce a current and subsequent surface potential [68]. The magnitude of this effect will depend on the application and relevant time scales.

### 2.2. Crossed-Beam Electron Scattering from Liquid Micro-Jets

**Figure 1:**
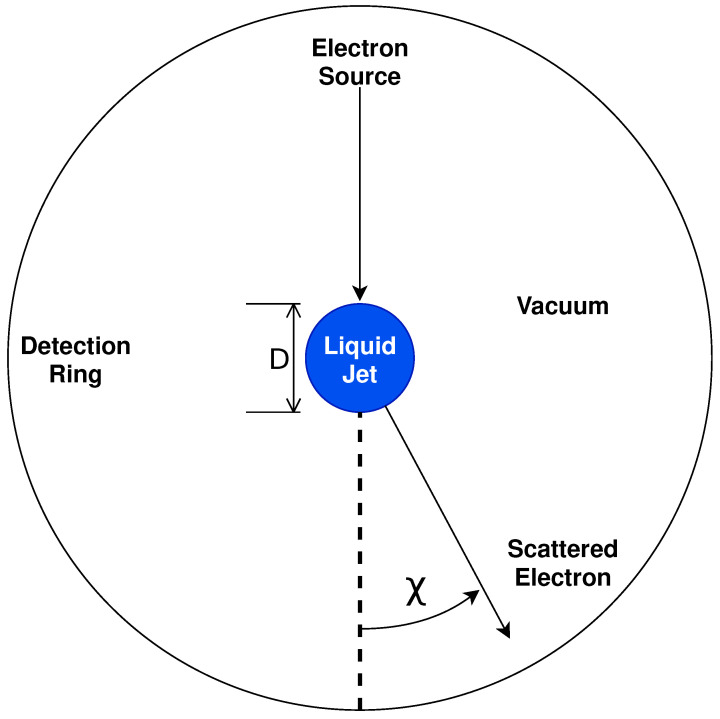
Diagram of crossed-beam electron scattering from a liquid micro-jet. The liquid is propelled into the page, while detectors are positioned normal to the liquid surface.

In our proposed experimental work, we will extend existing crossed-beam methodologies from gas to liquid environments, utilising liquid micro-jets to measure differential electron energy loss spectra (EELS), as shown schematically in Figure 1. The liquid would be propelled into a vacuum environment through a 15µm circular nozzle to produce a continuous, stable, and smooth scattering surface. In this work, we assume that the jet diameter is the same as the nozzle diameter. As noted previously, this jet remains stable for several millimetres before decaying into droplets. Evaporation at the surface and the tapering of the jet’s diameter are neglected in the current work, as the characterisation of such parameters was not available, and their effects on the model are expected to be minimal. Electrons, sourced from the thermionic emission from a tungsten filament, and collimated and transported by a series of DC-potential elements, are then propelled through the vacuum normal to the jet’s direction, to undergo multiple surface and bulk scattering events before escaping (and being collected by one or more detectors) or solvating within the liquid. For the foreshadowed work, an electron source with an energy full width at half maximum (FWHM) of 0.5eV will be used [74].

Once an electron escapes the jet, a scattered electron detector will resolve both its energy, ε, and scattering angle, χ, with a resolution of 0.8eV (FWHM) and $1^{\circ }$, respectively [74]. Through the detection of both the energy and angular dependencies, EELS provides a high degree of insight into the underlying electron scattering cross-sections and their associated dynamics. This combination of the electron gun and scattered electron detector provides a combined energy resolution of 0.9eV. We note that, with crossed-beam experiments, some restrictions will inevitably be placed on the available detection angles. This is simply due to the physical size of the electron source, the electron detector(s), and LµJ. Additionally, while an angle-resolved EELS provides sufficient information for the derivation of differential cross-sections within the gas phase, multiple scattering effects in liquids present significant challenges when deconstructing EELS into complete (differential) cross-section sets due to the high degrees of convolution arising. The extent of this convolution is investigated, in part, in Appendix B.

### 2.3. Extraction of Cross-Section Sets from EELS

The derivation of cross-sections from a reflected EELS measurement is akin to the ‘Inverse Milne Problem’, which consists of deriving the reflected EELS from a ‘half-plane’ medium, of finite or infinite depth from z=0 to z=L (where *L* is the half-plane depth), and extends to infinity in all other directions [75]. Outside the half-plane, a vacuum exists from which particles are propelled into the half-plane. The presence of high multiple-scattering at large *L*, relative to the scattering length, results in a complex combinatorial problem with a restricted set of observables, thanks to a reliance on external measurements [75].

The vast majority of treatments involve energy-independent radiative transfer [75,76] and neutron scattering [77,78]. The inclusion of energy dependencies was conceptually treated [79], with strict delta function requirements placed on both the source and detector, in order to derive total and differential cross-sections. In other studies, sufficiently thin (10–100nm) solid films were used to derive surface and bulk inelastic mean free paths [80,81,82], assuming a knowledge of the elastic cross-section. Likewise, cross-sections for amorphous ice films were derived under a two-stream assumption, along with a variation of film thickness between 0 and 5.456nm [16,83,84]. While the extension of these techniques towards LµJs may be feasible, other indirect approaches, such as Monte Carlo simulations, together with machine learning, could simplify the methodology significantly.

In multiple studies [41,42,43,45,46], machine learning was applied to the inversion of transport coefficients found through swarm experiments. While swarm transport coefficient measurements differ in nature to EELS, they each represent an ill-posed inversion problem associated with high degrees of multiple scattering. An extension of the current methodology towards liquid phase EELS requires a sufficiently large training dataset, which is facilitated through a highly configurable non-equilibrium Monte Carlo simulation of electron transport developed for this project. In this study, we thus seek an implicit cross-section extraction technique utilising a neural network trained through Monte Carlo simulations of an LµJ. In what follows, we outline the Monte Carlo simulation employed, and its application to the proposed LµJ measurements, before applying machine learning to this ill-posed inverse problem of determining cross-sections from LµJ-measured EELS.

## 3. Simulation of Electron Transport through Liquid Micro-Jets

Machine learning requires a substantial volume of training data to ensure a robust fitting process. To generate these data efficiently, a Monte Carlo simulation method was developed. Monte Carlo (MC) simulations provide flexible environments in which the manipulation of spatial and temporal parameters is both efficient and straightforward. LµJs inherently require specific and precise spatial variation in terms of the neutral density, and hence, an MC simulation is well suited for the task. The simulation technique is discussed in detail in [47], hence, here, we detail its application to LµJs, while a section of benchmark results is presented in Appendix A.

### 3.1. Liquid Dynamics

Relatively low-density gaseous environments involve an inherent assumption that each scattering event involves instantaneous localised interactions. In liquid environments, however, the de Broglie wavelength of the electron can be smaller than, or of the same order as, the inter-particle spacing and the mean free path, which necessitates modified coherent collision dynamics. To account for coherent scattering, a new method was recently developed which incorporated a structure factor modified cross-section [47]. Through this, the macroscopic effects of coherent scattering are realised, while retaining the efficiency of effective single particle scattering events. Therefore, the relative simplicity of modelling single particle-particle collisions is maintained, while replicating the macroscopic effects of multiple scattering. Through an integral structure factor [47,85], elastic collisions are split into three collisional processes, which are weighted by an angle-integrated structure factor, Γ(ε), produced through either theoretical or independent experimental techniques.

As a result of these microscopic processes, electron scattering through liquids is accurately simulated, given an appropriate structure factor. For real systems, experimentally measured structure factors are available through X-ray or neutron scattering [86,87,88]. While its functional form will vary, the methodology remains the same. Previously [47], it was shown that these processes match the required momentum and energy transfers derived using Boltzmann’s equation [47]. While isotropic scattering was assumed, the proof of principle was extended by the present authors to show its validity for the use of differential cross-sections. In the current work, we investigate structure effects upon measured spectra, while its implementation in our machine learning model is left for future study.

### 3.2. Interfacial Dynamics

In addition to liquid dynamics, interfacial effects must be considered in the simulation of LµJs. A recent study investigated density-dependent inter-facial effects on electron transport [89]. Due to a change in de-localised electron energy across the interface, a density-dependent ($n_{0}$), and hence spatially-dependent potential $V_{0}(n_{0}(\vec{r}))$, exists, which produces an effective electric field $E_{0}(\tilde{r})=-\nabla V_{0}(\vec{r})$. A spatial density dependency was thus implemented within the MC simulation, along with the resulting potential, and hence electric field. In that study [89], it was found that a discrete change in density, and hence potential, did not significantly change the path and associated transport dynamics of such particles when compared to a realistic functional form, which in addition to computational considerations, motivated the use of a step function form for the change in density in the current work. Additionally, any variation in the potential resulting from the density change is left for future extensions of this work.

### 3.3. Experimental Parameters

**Figure 2:**
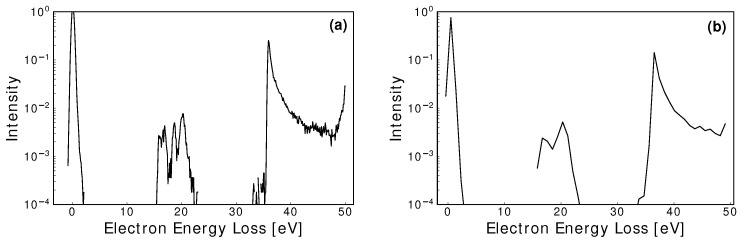
An example EELS comparison between a 0.1eV energy resolution (**a**) and a 0.9eV energy resolution (**b**), for an initial electron energy of 50eV scattering from neon [90]. Significant information is lost at 0.9eV, especially when the peaks are in close proximity.

Under realistic experimental conditions, detector resolutions and uncertainties will impact the prominence of correlations between EELS and their corresponding cross-sections. A critical factor for extracting important information is the experimental energy resolution of both the electron source and detector. In the models discussed above, an approximation to the energy spread of the electron source was incorporated by sampling a Gaussian distribution of FWHM of 0.5eV. In Figure 2, an experimentally feasible combined EELS energy resolution of 0.9eV [74], which is incorporated into the simulations in what follows in Section 4, alongside a hypothetical resolution of 0.1eV, are shown for comparison. Distinct information loss occurs in the 0.9eV energy-resolved spectra, for inelastic peaks with similar threshold energies, when compared to the 0.1eV spectra. This clearly indicates that the foreshadowed experimental measurements should be conducted with as narrow an energy resolution as possible.

### 3.4. Liquid Micro-Jet Parameters

To develop training data, simulated electrons were scattered through a cylindrical liquid micro-jet using the benchmarked MC simulation. Each electron, with initial energy $\varepsilon_{0}$, was fired along the *z*-axis towards a 15µm jet, which was directed along the *x*-axis. It was assumed that the motion of the jet (∼100$\;{\mathrm{ms}}^{-1}$) has a negligible effect on the measured EELS, as deflection angles were measured within the z-y plane and integrated over the *x*-axis.

Scattered electrons are detected once they reach a radial distance sufficiently far from the jet, such that the angle measured was relative to the jet’s centre in the z-y plane. The EELS was then integrated over the *x*-axis, such that there existed a $\left(\chi ,\varepsilon \right)$ dependence, where ε is the electron’s final energy, before integrating over χ. Each electron was simulated until it was detected, or until it lost sufficient energy, such that the probability of escape was negligible.

As a proof-of-concept for this work, we assume that the jet is gaseous neon at a liquid density of $2.13\times 10^{28}\;m^{-3}$, and at a temperature of 0K. Based on the proposed experiment [74], the energy variance of the initial electrons was sampled from a Gaussian with an FWHM of 0.5eV, centred around the initial energy $\varepsilon_{0}$, while its spatial extent was assumed to be a delta function directed along the surface normal. This serves as a first approximation to the experimental work, where there will be an electron beam, with a finite diameter of 1mm, incident on the LµJ.

## 4. Determining Cross-Sections from Electron Energy Loss Spectra Using Machine Learning

Through a sensitivity analysis, which is presented in Appendix B, we show that there exists a highly complex and degenerate correlation between the energy loss spectra and their underlying cross-sections. It was found that, while absolute magnitudes had little effect on the EELS, the effects of both relative magnitudes and energy dependencies of the cross-section were relatively significant. Deriving cross-section sets directly from EELS, without prior knowledge, is therefore a formidable task.

In this section, we apply machine learning to this ill-posed inverse problem of determining cross-sections from LµJ-measured EELS. As a proof-of-concept, we initially consider the task of determining electron–Ne cross-sections from EELS that are calculated using our Monte Carlo simulation. In what follows, we assume gas phase isotropic scattering, due to the present limited availability of liquid phase electron scattering and differential cross-sections, the inclusion of which remains a critical step towards the determination of liquid phase electron cross-sections. We note that, given appropriate liquid phase test data, the methodology that we propose here could be used to produce effective liquid phase electron cross-sections.

### 4.1. Machine Learning Methodology

To obtain a solution to the “inverse EELS problem”, we apply the same general machine learning approach as proposed by Stokes et al. [41,42,43,44], for the analysis of electron swarm transport data, and utilise an artificial neural network of the form:

(1) $$y\left(x\right)=\left(A_{4}\circ \mathrm{mish}\circ A_{3}\circ \mathrm{mish}\circ A_{2}\circ \mathrm{mish}\circ A_{1}\right)\left(x\right),$$

where $A_{n}\left(x\right)\equiv W_{n}x+b_{n}$ are affine mappings defined by dense weight matrices $W_{n}$ and *bias* vectors $b_{n}$, and $\mathrm{mish}\left(x\right)=x\tanh\left(\ln\left(1+e^{x}\right)\right)$ is a nonlinear activation function [91] that is applied element-wise. The output vector, y, contains each cross-section of interest:

(2) $$y=\left[\begin{array}{c} \sigma_{1}\left(\varepsilon \right)\\ \sigma_{2}\left(\varepsilon \right)\\ \vdots \end{array}\right],$$

all of which are a function of energy, ε, which becomes an input to the neural network alongside the available EELS data:

(3) $$x=\left[\begin{array}{c} \varepsilon \\ ineI_{1}\\ I_{2}\\ \vdots \end{array}\right],$$

where $I_{1},I_{2},\cdots$ are the electron intensities accumulated in each of the considered EELS energy bins. Note that we apply suitable logarithmic transformations to ensure that all inputs and outputs of the network are dimensionless and lie within $\left[-1,1\right]$. In what immediately follows, we specify that each bias vector contains 64 parameters, with the exception of $b_{4}$, of which the size must match the number of cross-sections in y. The weight matrices are sized accordingly.

In order to train the neural network, Equation (1), we require an appropriate set of example solutions to the inverse EELS problem. To ensure cross-sections provided by the network are physically plausible, we train on cross-sections from the LXCat project [11,12]. Specifically, we train with cross-sections of the form [42]:

(4) $$\sigma \left(\varepsilon \right)=\sigma_{1}^{1-r}\left(\varepsilon +\varepsilon_{1}-\varepsilon_{1}^{1-r}\varepsilon_{2}^{r}\right)\sigma_{2}^{r}\left(\varepsilon +\varepsilon_{2}-\varepsilon_{1}^{1-r}\varepsilon_{2}^{r}\right),$$

where $\sigma_{1}\left(\varepsilon \right)$ and $\sigma_{2}\left(\varepsilon \right)$ are a random pair of LXCat electron scattering cross-sections, sampled from the available targets, of a given type (e.g., excitation, ionisation, etc.), *r* is a pseudo-random number uniformly distributed between 0 and 1, and $\varepsilon_{1}$ and $\varepsilon_{2}$ are their respective threshold energies. Once suitable cross-sections are found for the training, corresponding LµJ EELS can be determined using our Monte Carlo simulation. In total, we consider 10,000 such training exemplars.

We implement and train the neural network, Equation (1), using the *Flux.jl* machine learning framework [92]. The network is initialised such that its biases are zero and its weights are uniform random numbers, as described by Glorot and Bengio [93]. Training is performed using the AdaBelief optimiser [94] with Nesterov momentum [95,96], step size $\alpha =10^{-3}$, exponential decay rates $\beta_{1}=0.9$ and $\beta_{2}=0.999$, and the small parameter $\epsilon =10^{-8}$. At each iteration, the optimiser is provided with a different batch of 4096 training examples, where each batch consists of 32 training cross-sections each evaluated at 128 random energies of the form $\varepsilon =10^{s}$, where $s\in \left[0,2\right]$ is sampled from a continuous uniform distribution. For each batch, the optimiser adjusts the neural network weights and biases with the aim of further minimising the mean absolute error in solving the inverse EELS problem for that batch. Training is continued for 250,000 iterations, providing an equal number of potential solutions to the inverse problem. We then select every 10 for the last 100,000 cross-sections, and the quality of each of these solutions is subsequently assessed by simulating their corresponding EELS and comparing those to Ne’s EELS to find the ‘best’ regression.

### 4.2. Cross-Section Regression Given the EELS

We now implement and train neural networks of the form of Equation (1), to determine a selection of Ne’s cross-sections given the corresponding EELS, while assuming full knowledge of Ne’s remaining cross-sections. In total, we use eleven EELS, corresponding to initial electron energies of 100, 79.43, 63.1, 50.12, 39.81, 31.62, 25.12, 19.95, 15.85, 12.59, and 10.0eV. In each spectra, a combined energy resolution of 0.9eV was assumed based on the detector performance that we currently find at Flinders University. Additionally, for computational considerations, each electron was simulated until 90% of its energy was lost. For the initial energies considered, no electron was simulated below 1eV, and thus we restrict the prediction domain to $\left[1\;\mathrm{eV},100\;\mathrm{eV}\right]$.

**Figure 3:**
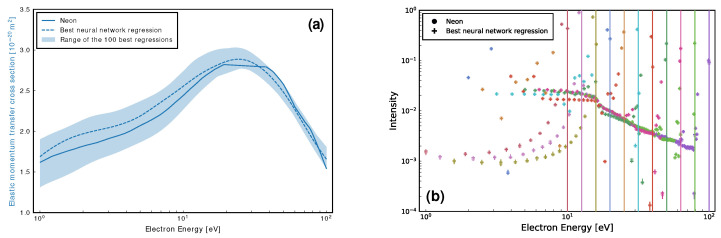
Neural network regression of Ne’s elastic MTCS [90], (**a**), and the corresponding agreement, (**b**), between its simulated EELS and the EELS used to perform the fit. The vertical lines in (**b**) denote the initial energies for each of the eleven EELS considered (from 10 eV to 100 eV incident energy, inclusive). Additionally, a shaded area in (**a**), along with vertical error bars in (**b**), are provided to indicate the range of the 100 best regressions. See also the figure legend for further details, noting that black is used to represent each colour of that marker shape and where colours correspond to each initial electron energies of 100, 79.43, 63.1, 50.12, 39.81, 31.62, 25.12, 19.95, 15.85, 12.59 and 10.0eV, indicated by vertical lines. In this model, a bias vector size of 64 was used, along with 250,000 iterations.

We first determine only Ne’s elastic momentum transfer cross-section (MTCS) from the considered EELS. Figure 3a shows a reasonable level of agreement between the elastic MTCS for Ne [90] and those found by the neural network, with the best regression seen to be accurate to within 9%. Figure 3b shows that, despite the large range in cross-sections for the 100 best regressions depicted in Figure 3a, the corresponding range of the EELS is much smaller, and in good agreement with those for Ne. This highlights the nonuniqueness of the inverse EELS problem, and suggests that there is not much room for improvement to the best cross-section fit plotted in Figure 3a unless; additional EELS were included, the energy resolution of the spectra was improved, or additional information about the unknown cross-sections was provided as input to the network.

**Figure 4:**
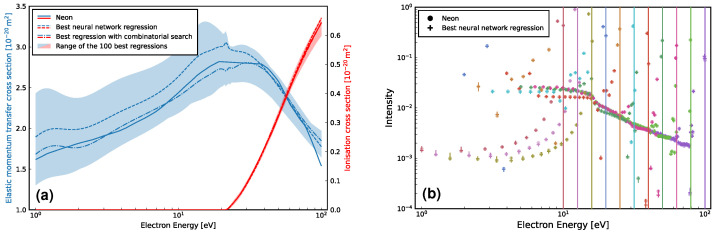
Simultaneous neural network regression of Ne’s elastic MTCS and ionisation cross-sections [90], (**a**), and the corresponding agreement, (**b**), between its simulated EELS and the EELS used to perform the fit. The vertical lines in (**b**) denote the initial energies for each of the eleven EELS considered (from 10eV to 100eV incident energy, inclusive). Additionally, a shaded area in (**a**), along with vertical error bars in (**b**), are provided to indicate the range of the 100 best regressions. See also the figure legends for further details, noting that black is used to represent each colour of that marker shape, where colours correspond to each initial electron energy of 100, 79.43, 63.1, 50.12, 39.81, 31.62, 25.12, 19.95, 15.85, 12.59, and 10.0eV, indicated by vertical lines. In this model, a bias vector size of 128 was used along with 300,000 iterations.

We follow the above by now simultaneously determining both the elastic MTCS and ionisation cross-sections for Ne, using the same set of EELS. In this model, the bias vector was increased to 128, while 300,000 iterations were performed. As expected, the uncertainty in the elastic MTCS has increased here, with a larger elastic MTCS envelope found in Figure 4a, with the best regression seen to be accurate to within 18%. In contrast, the corresponding ionisation cross-section envelope is particularly small, with the best regression being accurate here to within 1%. We attribute this high accuracy to the prominent ionisation “shoulder” present in six out of eleven of the EELS. Figure 4b shows that the corresponding EELS are now close in line with those for Ne, despite having a range that is slightly larger than their counterparts in Figure 3b.

Using the MC simulation, every combination of the best 100 elastic and ionisation cross-section pairs was compared through their resulting EELS to find a further improved cross-section set. While the maximum error in the elastic MTCS fit increased to 20%, the average error decreased resulting in a closer fit. For ionisation, the maximum error decreased to 0.2%, which further emphasises the remarkable predictive capability of this model for ionisation cross-sections.

The extension of this methodology to include the prediction of excitation cross-sections, or more specifically, the simultaneous prediction of three or more cross-sections, results in a substantial decrease in accuracy within the current methodology. Thus, the simultaneous prediction of elastic, ionisation, and excitation cross-sections is left for future iterations of this method, which should include advances in either the experimental resolution, or the fitting process.

## 5. Conclusions

We have developed a joint Monte Carlo and machine learning solution to the inverse Milne problem, that extracts electron cross-sections based on electron energy loss spectra from a micro-jet of dense gas. Machine learning was conducted using similar techniques outlined in the literature [41,42,43,44], while a new Monte Carlo simulation of a liquid micro-jet was developed. As a proof-of-concept, we found that the neural network determined neon’s gas phase elastic momentum transfer cross-sections to within 9%.

The extension towards the simultaneous determination of neon’s ionisation cross-sections, in addition to elastic scattering, decreased the accuracy to within 18% for elastic scattering, but was accurate within 1% for the ionisation cross-section. A combinatorial search was conducted using the 100 best elastic and ionisation cross-section pairs, which resulted in an accuracy to within 20% for the elastic MTCS and 0.2% for ionisation. While the maximum error for neon’s elastic MTCS increased, overall, the fit was improved, with only the higher energy regime suffering in accuracy. The determination of three or more simultaneous cross-sections resulted in a substantial decrease in accuracy, and is thus left for future iterations of this method.

As expected, absolute magnitudes were difficult to determine, although the prediction of the energy dependencies showed promise. Ionisation cross-sections were remarkably well determined, while the elastic cross-section prediction saw only quite slight discrepancies. Considering a relatively well-formed energy dependence, theoretical values at the higher energies could be used to ‘fix’ each cross-section to improve the current prediction. Alternatively (or in addition), the relative flow technique used for gas phase scattering experiments to achieve absolute cross-sections could be adapted to these liquid micro-jet experiments.

The main limitation with our current technique revolves around information density. Spectra are comprised of several ‘dead zones’, in which little to no scattering information exists. Additionally, a coarse energy resolution was shown to hide important information within the spectra, especially around inelastic peaks with similar threshold energies. To increase information density, a feature extraction algorithm might be utilised for each spectrum along with an improved experimental detector resolution. In other spectral studies, Gaussian de-convolution algorithms are employed to extract peaks from spectra. Utilising a similar approach, along with an increased detector resolution, one could provide further clarity to the network, assuming an appropriate treatment of asymmetry resulting from both multiple scattering and the ionisation energy sharing profiles.

Currently, a fitting algorithm is in development which, when applied to the method outlined in this study, aims to improve the predictive capability of the neural network. Additionally, the inclusion of appropriate liquid structure factors, surface potentials and space charge effects are necessary for real-world derivations of effective electron–liquid cross-sections. Overall, through a proof-of-concept model, we have shown that utilising machine learning, along with a significant wealth of Monte Carlo training data, one can reasonably predict individual and two simultaneous electron scattering cross-sections from EELS. The extension towards predicting full, self-consistent cross-section sets first begins with improvements on the methodology to ensure an improved accuracy in determining the cross-section magnitude.

## Figures and Tables

**Figure A1:**
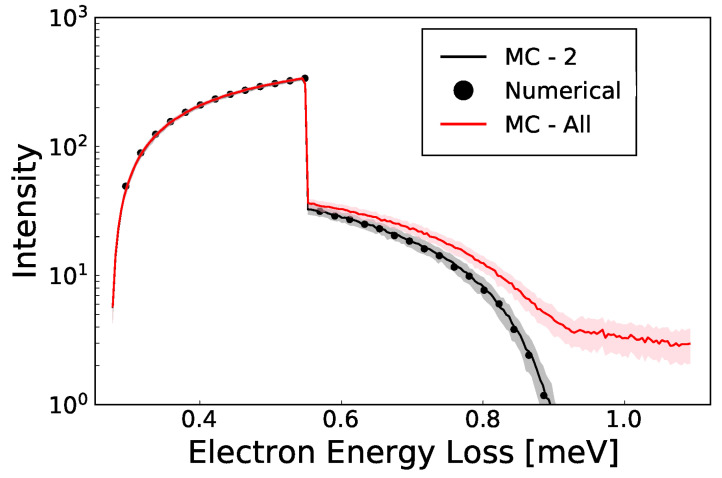
Comparison of the elastic peak in the EELS using a MC simulation including all collisions (red line), a MC simulation with 2 collisions (black line) and a Monte Carlo integration of 2 collisions (black dots). Shaded areas indicate the standard deviation of successive simulations. Resulting from single collisions, a large clear initial hump is present and consistent between all three models. A sharp drop-off followed by a decaying tail, then represents electrons that underwent multiple collisions. Excellent agreement was found between both the numerical and Monte Carlo method.

**Figure A2:**
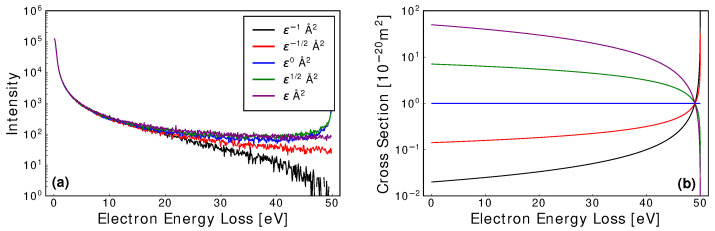
Electron Energy Loss Spectra (**a**), and their corresponding elastic cross-sections (**b**), for various power laws (see legend of (**a**)). An initial energy of 50eV was used. Each spectrum initially remains the same between each power law, before a divergence is observed at higher energy loss. Each spectrum for a single cross-section remains independent of the cross-section magnitude; however evident here is a dependence upon each cross-section derivative.

**Figure A3:**
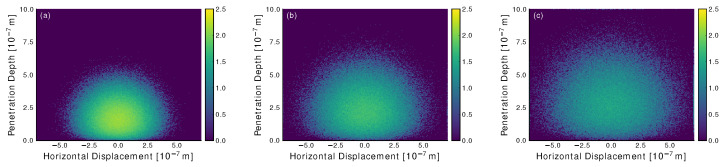
Heatmap of the electron cloud at $2.83\times 10^{-11}\;s$ after impact, with the LµJ surface for l=−1 (**a**), l=0 (**b**) and l=1 (**c**) according to Equation (A17). For this model, $10^{7}$1eV electrons were simulated through a 15µm LµJ with the jet directed into the page. The electron counts for each corresponding depth and horizontal displacement were then recorded. A $\log_{10}$ transform of non-zero values was then performed for visualisation purposes. Initially, in each scenario, the elastic cross-section is equal due to the initial energy of 1eV. In (**a**), the electron swarm loses energy through collisions with the background gas and experiences an increasing cross-section (decreasing mean free path) with energy loss, which results in a decreased expansion of the electron swarm. In (**b**), the electron swarm experiences a constant cross-section (constant mean free path) with energy loss, which results in a steady expansion of the electron swarm. Finally, in (**c**), the electron swarm experiences a decreasing cross-section (increasing mean free path) with energy loss, which results in an increased expansion of the electron swarm.

**Figure A4:**
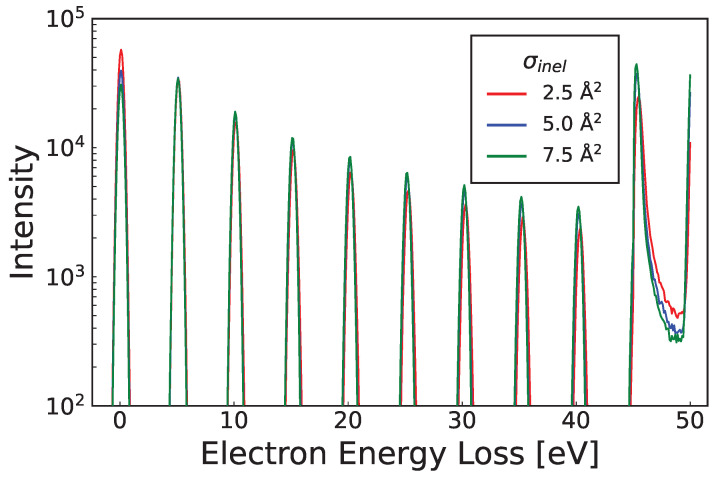
EELS for elastic and a single inelastic cross-section, with varying relative magnitude. An initial energy of 50eV was used, with an inelastic threshold of 5eV. The inelastic cross-section magnitude is shown in the legend. At 0eV energy loss an elastic peak exists, followed by threshold separated sequential inelastic peaks. Additionally, after 45eV of energy loss, electrons exclusively undergo elastic collisions which results in an increased intensity due to their minimal energy loss. Evident in this figure is a relative elastic to inelastic ratio dependence of the EELS upon the relative magnitude of the respective cross-sections.

**Figure A5:**
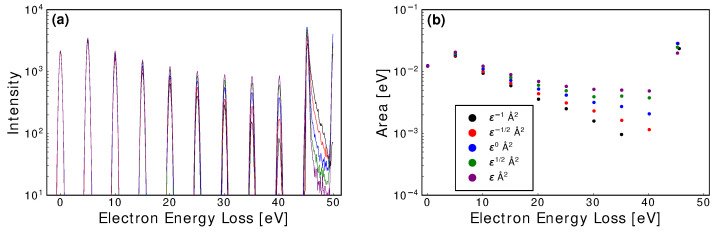
Simulated EELS (**a**), and area under each peak (**b**), as a function of the electron energy loss for a single elastic and inelastic cross-section, but both now with an equal power law functional form defined by the legend in (**b**). A distinct trend is observed, where the power law is proportional to the each cross-section’s slope.

**Figure A6:**
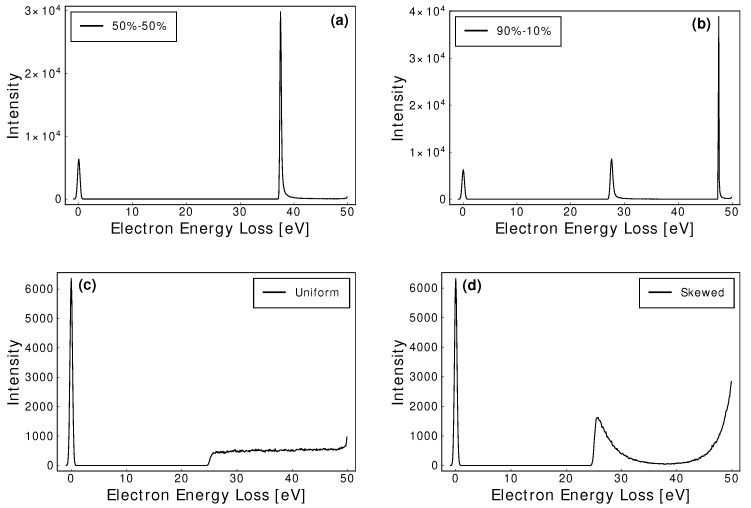
EELS for an elastic scattering and ionisation model. Initial energy was set to 50eV, with an ionisation threshold of 25eV. The energy sharing ratios, between the primary and secondary electrons, were set to 50–50% (**a**), 90–10% (**b**), uniformly randomised (**c**), and skewed randomisation in accordance to a normalised exponential cumulative distribution function (**d**).

**Figure A7:**
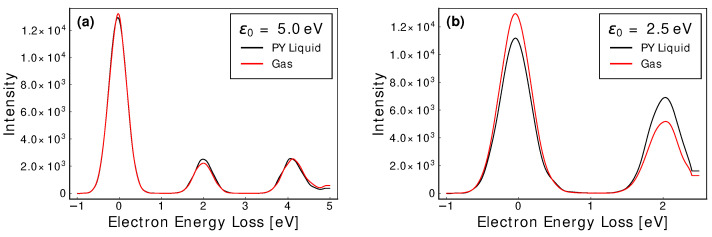
An elastic and inelastic EELS, with the Percus-Yevick liquid structure factor result (Black) compared with the equivalent gas phase result (Red). In each model, an inelastic threshold of 2eV was used with incident energies of 5 eV (**a**), and 2.5eV (**b**). As coherent scattering is predominantly restricted to low energies (<5 eV), its effects are minimal when the incident energies exist outside that domain.

**Figure A8:**
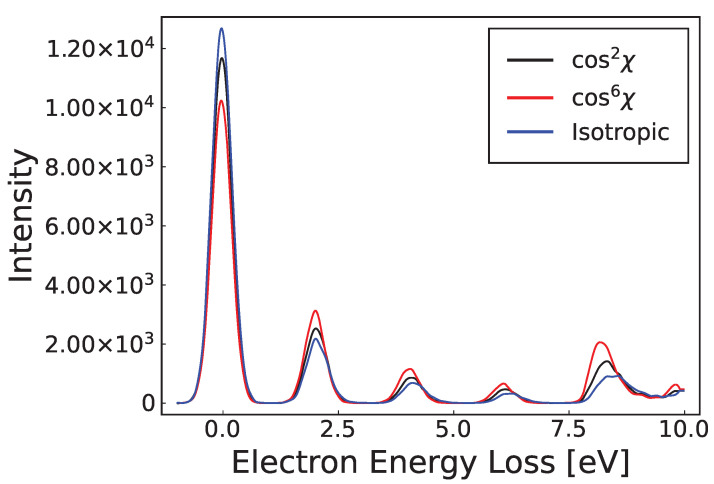
Elastic and inelastic EELS, with a varying differential cross-section. An initial energy of 10eV is used with an inelastic threshold of 2eV. Each DCS is represented using a normalised power of $\cos\left(\chi \right)$, as indicated in the legend. For higher degrees of forward scattering, a decrease/increase was observed at lower/higher energy loss.

## Appendix A. Benchmarking of the Liquid Micro-Jet Simulation

Throughout the development of the present MC simulation, systematic benchmarking was conducted through comparisons of transport coefficients for swarm experiments under highly non-equilibrium conditions (Appendix A.1). Furthermore, we have developed a relatively simple analytic model for the EELS, and have systematically benchmarked the simulation against this (Appendix A.2).

### Appendix A.1. Swarm Benchmarks

In this section, we first present Lucas and Saelee’s electron impact ionisation model to evaluate the accuracy of elastic, inelastic, and ionisation collisions [97]. We then present a modified Percus–Yevic benchmark [98], to benchmark the liquid scattering dynamics discussed in Section 3.1. Lucas and Saelee’s electron impact ionisation model is defined as the following:

(A1) $$\begin{array}{cc} \sigma_{elastic} & =4U^{-\frac{1}{2}}\;{Å}^{2},\\ \sigma_{inelastic} & =\left\{\begin{array}{cc} 0.1(1-F)(U-15.6)\;{Å}^{2}, & U\geq 15.6\;\mathrm{eV}\\ 0, & U<15.6\;\mathrm{eV} \end{array}\right.\\ \sigma_{ionisation} & =\left\{\begin{array}{cc} 0.1F(U-15.6)\;{Å}^{2}, & U\geq 15.6\;\mathrm{eV}\\ 0, & U<15.6\;eV \end{array}\right.\\ E/n_{0} & =10\;\mathrm{Td},\\ m/m_{0} & =10^{-3},\\ T_{0} & =0\;K, \end{array}$$

where *U* is energy in eV, *E* is the electric field, *m* is the electron mass, $n_{0}$ and $m_{0}$ are the background density and mass, respectively, while *F* is a parameter introduced to control the ratio of ionisation to inelastic collisions and reduced electric field, $E/n_{0}$, is given in Townsend units of $\mathrm{Td}=10^{-21}\;Vm^{2}$. This model’s strength comes from the fact that the total cross-section is independent of *F*. Therefore, variation of the ratio between ionisation and inelastic collisions allows for the isolation of the effects produced by each collision type. The results of this application are shown in Table A1 for *F* values of 0, 0.5 and 1, with excellent agreement being found between the Boltzmann equation and present MC results for each transport coefficient.

**Table A1:** Swarm benchmark results for Lucas and Saelee’s electron impact ionisation using the current MC code, presented alongside an independent solution to the Boltzmann equation [98]. Provided, for comparison, are the mean energy (ε), drift velocity (*W*) and the transverse ($D_{T}$) and longitudinal ($D_{L}$) diffusion coefficients for each value of *F*. Note that error estimates are given by the standard deviation of each transport coefficient with respect to time. For this benchmark $10^{6}$ particles were simulated for $n_{0}t=2\times 10^{16}\;{\mathrm{sm}}^{-3}$, where averages were conducted over the latter half of the simulation time.

F		ϵ	*W*	$n_{0}D_{T}$	$n_{0}D_{L}$
		[eV]	$\left[10^{4}\;{\mathrm{ms}}^{-1}\right]$	$\left[10^{24}\;m^{-1}s^{-1}\right]$	$\left[10^{24}\;m^{-1}s^{-1}\right]$
0	[98]	5.565	7.319	27.26	26.54
	Current	5.563	7.327	27.28	26.64
	Uncertainty	0.0001	0.003	0.03	0.04
0.5	[98]	5.224	8.593	27.26	28.65
	Current	5.223	8.594	27.23	28.62
	Uncertainty	0.001	0.005	0.04	0.07
1	[98]	4.969	9.474	27.23	29.33
	Current	4.968	9.487	27.25	29.42
	Uncertainty	0.002	0.008	0.05	0.01

We also present results from an adaptation of the Percus–Yevick model liquid benchmark, where the elastic cross-section is held constant and an additional, low energy inelastic cross-section is included:

(A2) $$\begin{array}{cc} \sigma_{elastic} & =6\;{Å}^{2},\\ \sigma_{inelastic} & =\left\{\begin{array}{cc} 0, & U<2\;\mathrm{eV}\\ 0.1\;{Å}^{2}, & U\geq 2\;\mathrm{eV} \end{array}\right.\\ \phi & =0,\;0.2,\;0.3,\;0.4\\ m_{0} & =4\;\mathrm{amu},\\ E/n_{0} & =3\;\mathrm{Td},\\ T & =0\;K, \end{array}$$

where ϕ is the so-called packing factor. For this model, a representative structure factor, detailed in [98], was implemented to verify coherent scattering. The time-averaged values are presented in Table A2, for various values of the packing factor ϕ, which is proportional to the density of the liquid used. Again, excellent agreement between the Boltzmann equation and present MC results, for each transport coefficient, is observed.

**Table A2:** Swarm benchmark results for our modified Percus–Yevik benchmark (Equation (A2)), from the current MC code, presented alongside an independent solution to the Boltzmann equation [98]. Provided, for comparison, are the mean energies (ε), drift velocities (*W*), and the transverse ($D_{T}$) and longitudinal ($D_{L}$) diffusion coefficients for each value of ϕ. Note that error estimates are given by the standard deviation of each transport coefficient with respect to time. For this benchmark $10^{5}$ particles were simulated for varying times, where averages were conducted over the latter half of the simulation time.

ϕ		ϵ	*W*	$n_{0}D_{T}$	$n_{0}D_{L}$
		[eV]	$\left[10^{4}\;{\mathrm{ms}}^{-1}\right]$	$\left[10^{24}\;m^{-1}s^{-1}\right]$	$\left[10^{24}\;m^{-1}s^{-1}\right]$
0	[98]	0.833	1.385		2.38
	Current	0.834	1.384	2.825	2.38
	Uncertainty	0.0001	0.001	0.017	0.02
0.2	[98]	0.976	3.397		6.32
	Current	0.977	3.388	9.09	6.35
	Uncertainty	0.0001	0.003	0.04	0.05
0.3	[98]	1.080	5.929		11.2
	Current	1.080	5.915	17.92	11.1
	Uncertainty	0.0001	0.001	0.009	0.01
0.4	[98]	1.233	10.52		19.51
	Current	1.234	10.50	34.97	19.51
	Uncertainty	0.0001	0.004	0.013	0.013

### Appendix A.2. Beam Benchmark

In this section, we derive a simplified analytic expression for results from reflected EELS measurements from an infinite half-plane given the underlying scattering cross-sections. We first note that, due to threshold excitation collisions, distinct peaks are expected to be observed in the EELS. The relationship between each scattering process (i.e., elastic, excitation and ionisation collisions) and their associated cross-sections, and the area of the corresponding EELS peak can be represented through an infinite series. Given the probability, $p_{n}$, of an electron escaping the jet after *n* collisions, the integral of an observed elastic scattering peak over the EELS, $I\left(\varepsilon \right)$, is given by the infinite series:

(A3) $$\begin{array}{cc} p_{1}\frac{\sigma_{el}\left(\varepsilon_{c}\right)}{\sigma_{t}\left(\varepsilon_{c}\right)}+p_{2}{\left(\frac{\sigma_{el}\left(\varepsilon_{c}\right)}{\sigma_{t}\left(\varepsilon_{c}\right)}\right)}^{2}+\\ p_{3}{\left(\frac{\sigma_{el}\left(\varepsilon_{c}\right)}{\sigma_{t}\left(\varepsilon_{c}\right)}\right)}^{3}+... & =\frac{1}{N}\int_{el}I\left(\varepsilon \right)d\epsilon , \end{array}$$

where the integration bound el is the peak width associated with pure elastic collision paths, ε is electron energy, $\sigma_{el}$ and $\sigma_{t}$ are the elastic and total cross-sections, respectively, and *N* is the initial number of electrons. For notation purposes, the left-hand side of Equation (A3) has been simplified as each subsequent collision will result in small energy losses that are encompassed by $\varepsilon_{c}$. Assuming a single collision, Equation (A3) reduces to:

(A4) $$p_{1}\frac{\sigma_{el}\left(\varepsilon_{0}\right)}{\sigma_{t}\left(\varepsilon_{0}\right)}=\frac{1}{I_{0}}\int_{el}I\left(\varepsilon \right)d\epsilon ,$$

where $\varepsilon_{0}$ is the initial energy. If we denote the unknown background medium with the subscript un, and introduce a reference medium ref, we can then use the ratio of their peaks to arrive at the following relationship:

(A5) $$\frac{\sigma_{elastic}^{un}\left(\varepsilon_{0}\right)}{\sigma_{total}^{un}\left(\varepsilon_{0}\right)}=\frac{p_{1}^{ref}}{p_{1}^{un}}\frac{I_{0}^{ref}\int_{el}I^{un}\left(\varepsilon \right)d\epsilon }{I_{0}^{un}\int_{el}I^{ref}\left(\varepsilon \right)d\epsilon }\frac{\sigma_{total}^{ref}\left(\varepsilon_{0}\right)}{\sigma_{elastic}^{ref}\left(\varepsilon_{0}\right)}.$$

Given the above relationship and assuming that $p_{1}^{un}=p_{1}^{ref}=1$, due to a restriction to single scattering events, such as with beam experiments in the gas phase, we can then derive unknown cross-section sets given the reference cross-section sets and the experimental limitations being considered. Attempts to extend this to include multiple collisions results in an ill posed set of equations [99]. However, under certain assumptions, limited analytical expressions can be derived, which are subsequently used for benchmarking the developed MC simulation package.

Equation (A3) can be used to derive an expression for each peak within the EELS, assuming we know the cross-section set and each probability $p_{n}$. Using a known cross-section, we thus seek an analytical or numerical solution for $p_{n}$. First, we consider the case for $p_{1}$. To escape, each electron must travel a distance $s_{1}$ without colliding, and then undergo a collision before travelling a distance $s^{\prime }$ towards the surface without undergoing another collision. If we assume a constant cross-section, the probability that a collision will occur at some distance *s* is given by the following:

(A6) $$p\left(s\right)ds=\frac{1}{l}\exp\left(-\frac{s}{l}\right)ds,$$

where *l* is the mean free path. Assuming now isotropic scattering, the probability of scattering with azimuthal angle χ and polar angle ψ, with respect to the initial velocity, is given by:

(A7) $$p\left(\chi \right)\sin\chi d\chi =\frac{1}{2}\sin\chi d\chi ,$$

where the ψ dependence has been integrated over and normalised. Each detected electron then escapes the jet by travelling a distance $s^{\prime }$, defined through:

(A8) $$s^{\prime }=-\frac{s}{\cos\chi }.$$

The probability to not collide in some distance $s^{\prime }$, is given by the cumulative distribution function (CDF):

(A9) $$\begin{array}{cc} P(s & >s^{\prime })=1-\int_{0}^{s^{\prime }}\frac{1}{l}\exp\left(-\frac{s^{\prime \prime }}{l}\right)ds^{\prime \prime }, \end{array}$$

(A10) $$\begin{array}{cc} P(s & >s^{\prime })=\exp\left(\frac{s}{l\cos\chi }\right). \end{array}$$

To couple the spatial and angular probability densities, we multiply Equations (A6), (A7) and (A10) together to obtain the probability that an electron will escape after a collision at distance *s* and angle χ:

(A11) $$P\left(\chi ,s\right)\sin\chi d\chi ds=\frac{1}{2l}\exp\left(-\frac{s}{l}\right)\exp\left(\frac{s}{l\cos\chi }\right)\sin\chi d\chi ds.$$

After performing a variable change to energy loss $u_{f}$, and utilising the following:

(A12) $$\frac{du_{f}}{d\chi }=\frac{2mm_{0}}{{\left(m+m_{0}\right)}^{2}}\sin\chi ,$$

where *m* and $m_{0}$ are the electron mass and target mass, respectively, then integrating Equation (A11) with respect to $\left(u_{f},s\right)$ we arrive at the expression for $p_{1}$:

(A13) $$p_{1}=\int \frac{1}{2}\left[\frac{{\left(m+m_{0}\right)}^{2}}{2mm_{0}}-\frac{1}{u_{f}}\right]du_{f}.$$

We then extend the method to calculate $p_{2}$, which requires the use of numerical integration techniques. Each electron now initially travels a distance $s_{1}$ and $s_{2}$, before each collision. The detected electron must then travel a distance $s^{\prime }$, towards the surface to escape. Note that the scattering angles χ and ψ, are now relative to the direction of motion prior to each respective collision. The probability that an electron will undergo each collision at $s_{1}$ and $s_{2}$, with scattering angles of $\left(\chi_{1},\psi_{1}\right)$ and $\left(\chi_{2},\psi_{2}\right)$, before escaping after some distance $s^{\prime }$, is given by:

(A14) $$P\left(s_{1},s_{2},s^{\prime },\chi_{1},\chi_{2},\psi_{1},\psi_{2}\right)=\frac{1}{16\pi^{2}l^{2}}\exp\left(-\frac{s_{1}}{l}\right)\exp\left(-\frac{s_{2}}{l}\right)\exp\left(-\frac{s^{\prime }}{l}\right),$$

where $s^{\prime }$ is represented through a similar CDF as Equation (A10). Assuming an initial position of $\vec{r}=\left[0,0,0\right]$, velocity $\vec{v}=\left[0,0,v_{z}\right]$, and a jet surface along $r_{z}=0$, each detected electron must return to the surface defined by $r_{z}=0$. To achieve an expression for this, we rotate the reference frame of the second collision by $\chi_{1}$, about an appropriate unit vector, to the initial frame of reference according to Rodrigues’ rotation formula. The *z* co-ordinate after two collisions is then given by:

$$r_{z}=s_{1}+s_{2}\cos\chi_{1}+s^{\prime }\left(\cos\chi_{1}\cos\chi_{2}-\sin\chi_{1}\sin\chi_{2}\cos\left(\psi_{1}-\psi_{2}\right)\right).$$

Setting $r_{z}=0$, we arrive at an expression for $s^{\prime }$:

$$s^{\prime }=-\frac{s_{1}+s_{2}\cos\chi_{1}}{\cos\chi_{1}\cos\chi_{2}-\sin\chi_{1}\sin\chi_{2}\cos\left(\psi_{1}-\psi_{2}\right)}.$$

Substituting this into Equation (A14) we find:

(A15) $$\begin{array}{cc} P\left(s_{1},s_{2},\chi_{1},\chi_{2},\psi_{1},\psi_{2}\right)= & \frac{1}{16\pi^{2}l^{2}}\exp\left(-\frac{s_{1}}{l}\right)\exp\left(-\frac{s_{2}}{l}\right)\\ \; & \times \exp\left(\frac{1}{l}\frac{s_{1}+s_{2}\cos\chi_{1}}{\cos\chi_{1}\cos\chi_{2}-\sin\chi_{1}\sin\chi_{2}\cos\left(\psi_{1}-\psi_{2}\right)}\right). \end{array}$$

The integration of Equation (A15) over the appropriate limits, along with a transformation of variables to energy loss, gives $p_{2}$. Any non-physical limits were removed using step functions, and they are detailed in what follows. First, bounds for which $s^{\prime }<0$ were removed as if $s^{\prime }<0$ the electron has already escaped after a single collision and is accounted for in $p_{1}$. Additionally, any divisions by 0 were also removed. Extending this methodology to higher terms is theoretically possible. However, in this investigation we include only $p_{1}$ and $p_{2}$ due to limitations in the available computation power.

For illustrative purposes and a comparison, a MC simulation of a 15µm LµJ was then considered with the following model:

(A16) $$\begin{array}{cc} \sigma_{elastic} & =1\;{Å}^{2},\\ \sigma_{inelastic,1} & =\left\{\begin{array}{cc} 0, & \epsilon <0.3\;\mathrm{eV}\\ 1\;{Å}^{2}, & \epsilon >0.3\;\mathrm{eV} \end{array}\right.,\\ \sigma_{inelastic,2} & =\left\{\begin{array}{cc} 0, & \epsilon <0.5\;\mathrm{eV}\\ 1\;{Å}^{2}, & \epsilon >0.5\;\mathrm{eV} \end{array}\right.,\\ n_{0} & =2.13\times 10^{28}\;m^{-3}. \end{array}$$

Additionally, a numerical integration (using a Monte Carlo technique) was conducted for up to two collisions, and compared with the simulated results in Figure A1. MC simulations that measure both all and up to 2 collisions are included as separate series. Also included as shaded regions are error estimations based upon standard deviations between successive simulations. Excellent agreement was found for up to two collisions. While being somewhat limited in scope, this benchmarking further lends confidence to the accuracy of the simulation.

## Appendix B. Electron Energy Loss Spectra: Sensitivity to the Scattering Dynamics and Experimental Parameters

In this study we seek an implicit relationship between the EELS and its underlying cross-section set through the use of demonstrative cross-section sets. In what follows we now systematically investigate the ramifications of individual changes to the cross-section sets upon the EELS. For the conditions under investigation, no electron transmission through the beam of 15µm was observed.

### Appendix B.1. Magnitude and Energy Dependence of Elastic and Excitation Cross-Sections on the EELS

#### Appendix B.1.1. Effect of the Elastic Cross-Section Dependence on the EELS

In order to de-convolute features present within the EELS and their relationship with the underlying cross-section set, each parameter within the model was isolated and tested for its effect upon the EELS. The first model considered was a constant elastic cross-section:

$$\begin{array}{cc} \sigma_{elastic} & =\sigma_{0}\;{Å}^{2}, \end{array}$$

where $\sigma_{0}$ was varied to determine the impact of the cross-section magnitude on the simulated EELS. As expected, an invariance with respect to magnitude was observed as no transmission occurred and thus cross-section magnitude changes will scale the problem without affecting the reflected intensities. The second elastic model considers the effect of the energy dependence of the cross-section. Specifically, in Figure A2 we compare the effect of various power laws, through the elastic only model:

(A17) $$\begin{array}{cc} \sigma_{elastic} & =\varepsilon^{l}\;{Å}^{2}, \end{array}$$

where ε is energy in eV and *l* is varied in accordance with the legend of Figure A2. Each spectrum showed an invariance with respect to the power law at low energy losses, and exhibited intensity decay rates at higher energy loss that were dependent upon the power law used. The magnitude of this effect depends on the energy dependence of the cross-section’s derivative, and gradual changes were found to have a minimal impact upon the spectra.

To explain this physically, one can imagine the swarm of electrons as an expanding sphere in Cartesian space. Figure A3 shows the electron swarm within the jet at time $2.83\times 10^{-11}\;s$ after impact with a LµJ for $l\in \left[-1,0,1\right]$. A constant mean free path with energy loss (l=0) results in considerable back scattering after jet surface impact, and the remaining electron cloud travels further inward with a constant expansion rate. Nothing inherently changes during their flight and the back-scattering flux is proportional to the number of remaining electrons within the jet. A decreasing mean free path with energy loss (l=−1) results in a diminishing rate of expansion, that results in a relatively smaller back-scattered intensity. In contrast to this, an increasing mean free path with energy loss (l=1) accelerates the rate of expansion, resulting in an increased likelihood of back scattering out of the jet.

#### Appendix B.1.2. Effect of the Excitation Cross-Section Dependence on the EELS

Excitation cross-sections are introduced through the model:

$$\begin{array}{cc} \sigma_{elastic} & =10\;{Å}^{2},\\ \sigma_{excitaiton} & =\left\{\begin{array}{cc} 0, & \varepsilon <5\;\mathrm{eV}\\ \sigma_{0}\;{Å}^{2}, & \varepsilon >5\;\mathrm{eV} \end{array}\right., \end{array}$$

where $\sigma_{0}$ is varied to investigate the relative elastic/excitation cross-section magnitudes and their effects on the EELS. As shown in Figure A4, it is clear that the intensity peak at 0eV energy loss is proportional to the elastic cross-section. Inelastic peaks are then observed at intervals equal to the 5eV threshold energy. Once the energy of the electrons falls below that threshold, energy loss is minimal due to the restriction to elastic collisions. This results in a high energy loss peak that is proportional to the elastic mean free path. Additionally, as the inelastic cross-section magnitude increases, the below threshold peak at 45eV energy loss also increases due to an increased energy loss rate. Note that while the initial energy in all cases is sampled from a Gaussian, energy loss is defined as the difference between the average initial energy and final energy, which can result in an apparent negative energy loss in some cases.

As expected, the relative elastic/excitation cross-section magnitude is correlated with each peak found in the EELS. As shown previously, in a single collision regime and within an ideal environment, direct relationships exist between the two quantities. However, the introduction of multiple scattering convolutes their dependence. While a clear trend also exists here, excitation processes within a multiple scattering environment introduce peak dependencies upon relative cross-section magnitudes at multiple energies and results in a large combinatorial problem.

Shown in Figure A5, we expand the previous model to include energy dependencies with multiple cross-sections through the following model:

$$\begin{array}{cc} \sigma_{elastic} & =\varepsilon^{x}\;{Å}^{2},\\ \sigma_{inelastic} & =\left\{\begin{array}{cc} 0, & \varepsilon <5\;\mathrm{eV}\\ \varepsilon^{x}\;{Å}^{2}, & \varepsilon >5\;\mathrm{eV} \end{array}\right., \end{array}$$

where *x* is varied according to the legend of Figure A5. No change in the elastic peak at 0eV is observed, however each subsequent peak decays at a rate dependent upon the power law used.

For comparison, integration over the fitted exponential modified Gaussians for each peak are shown in the right hand graph of Figure A5 (note that for x=−1, the 40eV peak was not found by the fitting algorithm). In contrast to the single elastic cross-section result outlined in Appendix B.1.1, a distinct correlation was found. The introduction of an inelastic process dramatically increases the energy loss rate, which in turn will amplify the effects of an energy-dependent cross-section. A direct relationship remains convoluted, however, as the magnitude and energy dependence of the individual cross-sections, within a given cross-section set, will vary, resulting in significant degeneracy issues.

Of particular interest is the below threshold peak at 45eV in the EELS. Here it was found that while their areas remain relatively constant, there exists a relationship between their asymmetric nature and the power law used. The exact nature of the relationship depends upon each cross-section’s, and hence mean free path’s, derivative with respect to energy. As discussed in Appendix B.1.1, when compared to scattering with a constant mean free path, an increasing mean free path with each subsequent collision increases the expansion rate for the electron swarm due to the reduced collisional impediment, resulting in a larger scattered intensity. As the swarm falls below the threshold energies, where energy loss is minimal, fewer electrons remain in the liquid when compared with the result for the constant mean free path model and thus a decreased scattered intensity was observed. A decreasing mean free path with energy loss slows the rate at which the swarm expands, which results in a reduced intensity when compared to a constant mean free path. As the mean free path decreases even further at lower energies, the probability of escape reduces even further, which effectively traps the electron swarm within the liquid.

#### Appendix B.1.3. Effect of the Ionisation Cross-Section Dependence on the EELS

To investigate ionisation collisions, and their effect upon the EELS, the following model was used:

$$\begin{array}{cc} \sigma_{elastic} & =1\;{Å}^{2},\\ \sigma_{ionisation} & =\left\{\begin{array}{cc} 0, & \varepsilon <25\;\mathrm{eV}\\ 1\;{Å}^{2}, & \varepsilon >25\;\mathrm{eV} \end{array}\right., \end{array}$$

with varying ionisation energy-sharing profiles between the primary and secondary electrons as shown in Figure A6. At this point, it is important to consider the effects of fractional energy loss on the multiple scattering features of the EELS. Energy loss is largely driven by threshold excitations, energy partitioning losses (i.e., distribution of the excess energy between the scattered and ejected electrons) and kinetic energy exchange between the target and the electron (see Equation (A12)). Energy exchange losses are proportional to the incident energy of the electron (and the mass ratio with the target atom/molecules), and result in a narrowing of the features (i.e., a reduction in average energy loss) at low energies.

While excitation collisions introduce relatively simple peaks within the spectra, ionisation collisions introduce unique features depending upon the energy-sharing profile between the primary and secondary electrons. Four model scenarios were considered (see Figure A6; equal, biased, uniform and skewed sharing). Equal energy sharing (i.e., a 50–50% energy partition) between the primary and secondary electrons resulted in a single peak at an energy loss of the ionisation threshold plus half the ionisation threshold energy. Once below the energy threshold, electrons undergo elastic collisions at minimal energy loss resulting in a relatively large ionisation peak. Biased energy sharing was represented through a 90–10% sharing partition, resulting in separate peaks that exhibit broadening due to energy exchange losses. While the observed amplitudes and full width half maximums vary according to the aforementioned narrowing effect, the total peak area was equal in accordance with the equal cross-sectional magnitudes used (see Figure A6).

As expected, a uniform probability of all sharing ratios resulted in a shoulder beginning at the ionisation threshold energy loss. A gradual increase was observed in the shoulder intensity once again, due to the energy exchange losses discussed above. Finally, a normalised exponential cumulative distribution function was utilised to emulate a skewed energy sharing distribution for the ionisation process. A semi-symmetric saddle shape was observed, with an asymmetry again driven through energy exchange losses.

### Appendix B.2. Anisotropy in the Scattering Dynamics

#### Appendix B.2.1. Coherent Scattering Effects

As discussed previously, modelling coherent scattering in liquid environments was achieved through the addition of liquid structure factors [47,85]. Figure A7 compares how those coherent liquid scattering structure factors affect the present EELS utilising the model:

$$\begin{array}{cc} \sigma_{elastic} & =6\;{Å}^{2},\\ \sigma_{inelastic} & =\left\{\begin{array}{cc} 0, & \varepsilon <2\;\mathrm{eV}\\ 0.1\;{Å}^{2}, & \varepsilon >2\;\mathrm{eV} \end{array}\right., \end{array}$$

with the Percus-Yevick structure factor [47]. Coherent scattering effects are isolated to the low energy regime (<5 eV), and hence its effects were limited outside that range. At low energies, electrons tend to coherently scatter through multiple targets, with little angle change, and hence will travel further in a particular direction when compared to isotropic scattering binary collisions. It was found that initial energies below 5eV resulted in a decreased surface scattering intensity, due to enhanced forward scattering. Multiple scattering then randomises the preferred direction, such that the swarm will expand in size faster than the isotropic equivalent, which resulted in an increased intensity observed at the higher energy loss.

#### Appendix B.2.2. Differential Cross-Section Effects

Anisotropic scattering was included through a differential cross-section (DCS) in the following model scenario:

$$\begin{array}{cc} \sigma_{elastic} & =6\;{Å}^{2},\\ \sigma_{inelastic} & =\left\{\begin{array}{cc} 0, & \varepsilon <2\;\mathrm{eV}\\ 0.1\;{Å}^{2}, & \varepsilon >2\;\mathrm{eV} \end{array}\right., \end{array}$$

where varying degrees of forward scattering were considered and represented through a normalised powers of the cosχ function, where χ is relative to the incident direction of motion. Each modified DCS was applied to both the elastic and inelastic cross-sections.

Similar to coherent scattering effects, an initial decrease in intensity at 0eV energy loss was observed in Figure A8 along with an increased signal at higher energy loss that is proportional to the degree of forward scattering. The explanation of that latter observation is the same as in Appendix B.2.1, except now it is applicable to all incident energies.
